# The Epidemiological Trend of Acute Myeloid Leukemia in Childhood: a Population-Based Analysis

**DOI:** 10.7150/jca.32326

**Published:** 2019-08-27

**Authors:** Xuanwei Chen, Jianwei Pan, Shuncong Wang, Shandie Hong, Shunrong Hong, Shaoru He

**Affiliations:** 1 Department of Pediatric, Guangdong Provincial People's Hospital and Guangdong Academy of Medical Science, Guangzhou 510080, Guangdong, China; 2 Theragnostic Laboratory, Department of Imaging and Pathology, Biomedical Sciences Group, KU Leuven, Herestraat 49, Leuven 3000, Belgium; 3 Department of Neonatal Intensive Care, Chaozhou People's Hospital, Chaozhou 521000, Guangdong, China; 4 Department of Radiology, Puning People's Hospital, Puning 515300, Guangdong, China

**Keywords:** AML, incidence, survival, children, period analysis

## Abstract

Acute myeloid leukemia (AML) is the fifth most common malignancy in children, and the prognosis for AML in children remains relatively poor. However, its incidence and survival trends based on a large sample size have not been reported.

Children diagnosed with AML between 1975 and 2014 were accessed from the Surveillance, Epidemiology, and End Results database. Incidence and survival trends were evaluated by age-adjusted incidence and relative survival rates (RSRs) and Kaplan-Meier analyses. Cox regression was performed to identify independent risk factors for child AML death.

The overall incidence of AML in childhood increased each decade between 1975 and 2014, with the total age-adjusted incidence increasing from 5.766 to 6.615 to 7.478 to 7.607 per 1,000,000 persons. In addition, the relative survival rates of AML in childhood improved significantly, with 5-year RSRs increasing from 22.40% to 39.60% to 55.50% to 68.30% over the past four decades (*p* < 0.0001). Furthermore, survival disparities among different races and socioeconomic statuses have continued to widen over the past four decades. Multivariate Cox regression analyses showed a higher risk of death in Black patients (HR = 1.245, 95% CI: 1.077-1.438, *p* = 0.003) with Whites as a reference.

These results may help predict future trends for AML in childhood, better design clinical trials by eliminating disparities, and ultimately improve clinical management and outcome.

## Introduction

Leukemia covers a group of cancer that represents abnormal cells originating from the hematopoietic system with poor differentiation and aggressiveness 1. Leukemia has long been the second most common cause of cancer-related death in patients younger than 39 years 2. According to estimation, 61,780 new leukemia cases and 22,840 leukemia-specific deaths will occur in the U.S.A. in 2019 3. Acute myeloid leukemia (AML) is a malignancy originating from hemopoietic stem cells by clonal expansion of abnormally differentiated blasts of myeloid lineage 4. A previous report showed that AML usually occurred in elderly adults, of whom the median age was 67 years, and approximately 30% of AML patients were over 75 years 5. With the developments of hematopoietic stem cell transplantation, chemotherapy and supportive care, a great improvement in survival has been observed for AML over the past decades. Advances in the elucidation of alterations in genetics may help prompt patient survival, especially in APL 6, 7. In addition, the development of chemotherapy and hematopoietic stem cell transplantation increases the clinical management of relapse cases 8. Given these changes in the clinical management of AML, it will be important to perform quantitative epidemiological analyses on survival trend.

Although the 5-year survival rates for malignancy in children have increased to approximately 80% with developments in diagnosis and treatment, leukemia is still the second leading cause of death in children aged between 5 and 14 years 9. Among them, AML is ranked the fifth most common malignancy in children, and the prognosis for AML in children (chAML) remains relatively poor, with a 5-year survival rate of 64% compared with 90% in acute lymphoid leukemia 10. In addition, accumulating evidence has demonstrated that AML is characterized by its heterogeneity among age groups, sexes, and races, and whether this heterogeneity can be observed within children remains unknown 11. To demonstrate the epidemiological trend of chAML, we used period analysis methodology to evaluate changes in patient survival over the past four decades. It has been proven that a period analysis provides more precise predictions of survival than a traditional cohort analysis 12-14.

In addition, racial and socioeconomic status (SES) inequalities continue to increase in the healthcare system, and more importantly, the eye-popping gaps have been confirmed to be associated with survival imbalance among cancer patients 15, 16. Hence, we analyzed the general trends of incidence and survival as well as the survival trends by race, age, and SES via a population-based database in the U.S.A.

## Materials and Methods

### Case inclusion

We accessed Surveillance, Epidemiology, and End Results (SEER) cancer registry data, which are maintained and updated by the National Cancer Institute, for AML cases. For all incident cancer cases in their coverage areas, the SEER registries collect clinicopathological and demographic information, including sex, race, age, socioeconomic status (SES), survival time, and vital status 17. The SEER registry is considered an accurate representation of the U.S. population with cancer, with 18 SEER registry sites covering approximately 28% of the total population and nine sites covering 9% of the total population 18.

Data collection was performed by SEER*Stat version 8.3.2. Cases of acute myeloid leukemia (AML) were histologically identified by the following ICD-O-3 codes: 9840/3, 9860/3, 9861/3, 9865/3, 9866/3, 9867/3, 9869/3, 9870/3, 9871/3, 9872/3, 9873/3, 9874/3, 9891/3, 9895/3, 9896/3, 9897/3, 9898/3, 9910/3, 9911/3, 9920/3, and 9931/3. Children were selected by restricting the age at diagnosis (< 15 years). We identified patients with first primary AML diagnosed between 1 January, 1975 and 31 December, 2014. Cases diagnosed between 1975 and 2014 were analyzed with each decade as one unit. We excluded patients who had metastatic disease, who was diagnosed by a death certificate or autopsy report and whose age, race, SES status or survival time was unknown.

Incidence data from nine registry sites by each calendar year and decade were extracted to maintain the patient resource over the study period. Relative survival rate (RSRs), calculated by SEER*stat software, has been widely used to demonstrate the net survival of any specific cancer. To reduce the confounding effect of age while comparing the incidence rates of different patient groups, we calculated age-adjusted incidences, which are weighted by the proportions of persons in the corresponding age groups of a standard population. The 2000 U.S. standard population was selected as the standard population in the current study. Incidence rates in the current study are expressed as per 1,000,000 persons. Relative survival rates were calculated by dividing the number of surviving patients at a given time point after diagnosis by the number of persons in the same category that had not been diagnosed with AML. In addition, all-cause survival was also performed. Incidence and survival analyses were performed after patients were categorized into groups by sex, SES, race, and age. The county-level SES was used to indicate its residents' SES statues as previously reported 19.

### Statistical analyses

The all-cause survival difference was evaluated by Kaplan-Meier method with log-rank test for statistical examination. The risk factor for all-cause death was assessed by Cox regression. Specifically, factors that are significantly related to the survival in univariate Cox regression are inputted into multivariate Cox regression 20. These statistical analyses were performed based on survival package in R 3.3.3 21, 22. A two-tailed *p*-value less than 0.05 was defined as significant. All figures were made by GraphPad 7.0.0.

## Results

### Incidence trend of AML in children over the past four decades

To better unify the population studied over the past four decades, incidence data for the original nine SEER registries were accessed. As shown in Table 1, a total of 1490 AML cases were identified in children (chAML), with 288, 342, 424 and 436 cases in each decade. In general, 48% of patients aged between 0 and 4 years and this proportion continued to increase in each decade, with 40%, 48%, 50%, and 52% respectively. The number of chAML in both sexes are similar in each decade, with a slightly higher number in the male. In terms of race, 71% of patients were Whites in general, and this proportion decreased in each decade due to the increasing percentage in Blacks. In terms of socioeconomic status (SES), patients from low-poverty regions accounted for approximately 54%, compared with 43% in medium-poverty regions and 3% in high-poverty regions.

**Figure 1:**
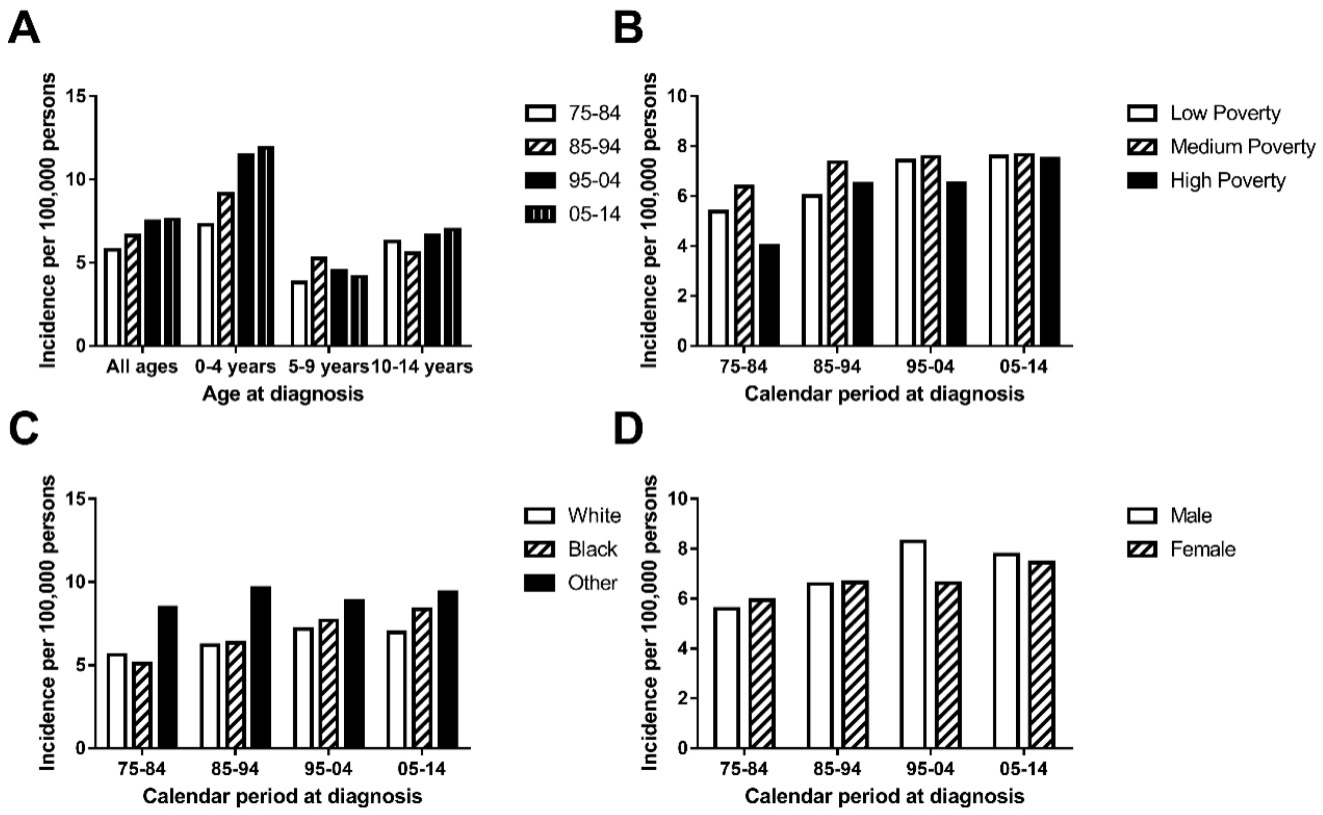
Incidence (A.) of AML cases are shown by age group (total and ages 0-4, 5-9 and 10-14) and calendar period. Incidence (B, C, D.) of chAML cases in each decade are grouped by SES, race and sex respectively.

The general incidence of chAML in the first decade (5.766 per 1,000,000) was lower than that in the second decade (6.615 per 1,000,000), which was also lower than that during 1995-2004 (7.478 per 1,000,000) and 2005-2014 (7.607 per 1,000,000) (Figure 1A and Supplementary Table 1). This similar trend was also found in a patient group aged between 0 and 4 years. However, this increasing trend over time was not observed in the patient group aged between 5 and 9 years and between 10 and 14 years, who showed a relatively stable and fluctuating pattern. More interestingly, the highest incidence rate was observed in patients aged 0 and 4 years in each decade. The AML incidence per 1,000,000 continued to increase in the age group between 0 and over 4, with incidence rates of 5.766, 6.615, 7.478 and 7.607 in each decade (Figure 1A).

The medium-poverty group showed the highest incidence rate, with incidence rates of 6.375, 7.365, 7.577 and 7.65 per 1,000,000 persons in each decade, which remained relatively stable over time. Both the low-poverty and high-poverty groups showed increasing incidence over time, and the low-poverty group (with incidences of 5.383, 6.000, 7.42 and 7.595 per 1,000,000 persons in each decade) distinguished itself by reaching an incidence plateau 10 years ahead of that of the high-poverty group (Figure 1B). In terms of race, Others showed the highest incidence, with a fluctuating incidence pattern over time (8.458, 9.639, 8.884 and 9.393 per 1,000,000 persons in each decade). Whites and Blacks showed similar incidences in each decade and shared a slightly increasing pattern (Figure 1C). In addition, the incidences were similar between sexes in each decade (5.598 vs. 5.943 in 1975-1984, 6.586 vs. 6.645 in 1985-1994, 8.297 vs. 6.619 in 1995-2004 and 7.762 vs. 7.444 in 2005-2014; Figure 1D).

### Survival changes in AML over time

**Figure 2:**
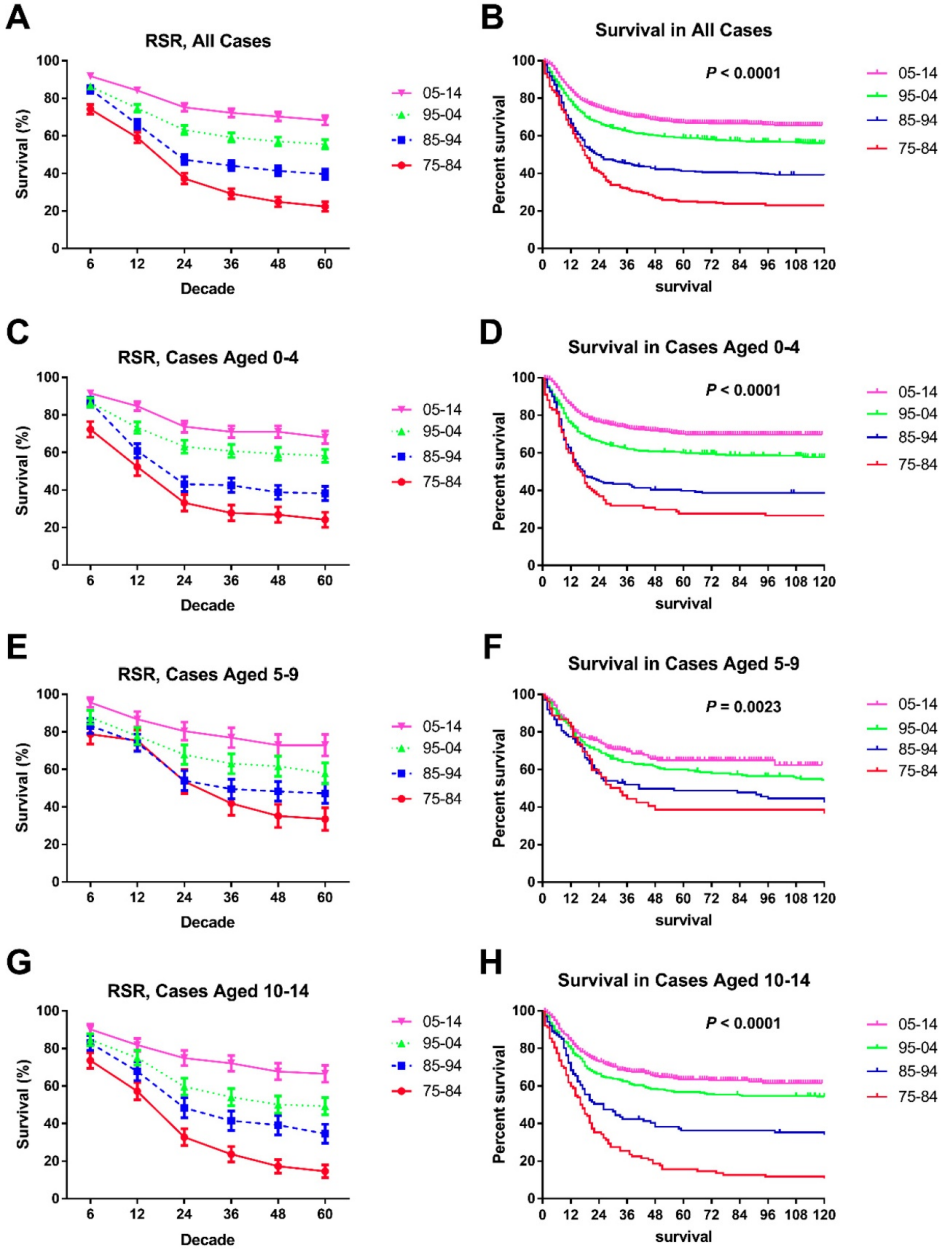
Trends in 5-year relative survival rates and all-cause survival by Kaplan-Meier survival analysis for patients diagnosed with chAML at nine SEER sites between 1975 and 2014 according to age group (total and ages 0-4, 5-9 and 10-14 years) and calendar period.

Survival of chAML increased each decade, with median survival times of 18 and 24 months in the first two decades, and the median survival in the last decades was longer than 60 months. These increasing survival trends were further verified by both RSRs and Kaplan-Meier method in all chAML cases and in each age group (*p* < 0.0001 for both) (Table 2 and Figure 2). For instance, the 6-month RSRs improved from 74.20% to 84.80% to 86.30% to 91.80%, and a greater increment was observed in early decades. More importantly, survival increment is more obvious in long-term survival than short-term survival (Table 2). For instance, the 5-year RSRs improved from 22.40% to 39.60% to 55.50% to 68.30% over time. This survival increment advantage in long-term survival was also seen in every age group. Of note, the survival improvement in patients aged between 5 and 9 years over time was less obvious, with 6-month RSRs improving from 78.7% to 83.2% to 87.7% to 95.6% (Table 2).

**Figure 3:**
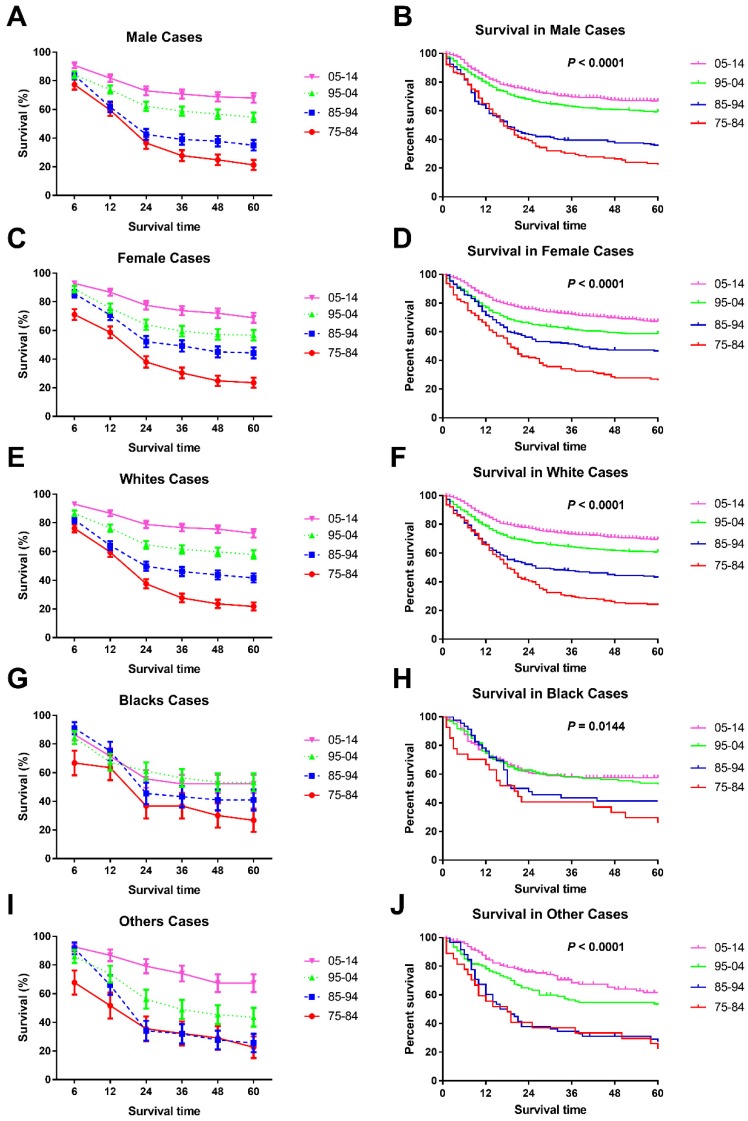
Trends in 5-year relative survival rates (A, C, E, G, I.) and all-cause survival by Kaplan-Meier analyses (B, D, F, H, J.) by sex and race in children patients diagnosed with chAML between 1975 and 2014 at the original nine SEER sites.

Significant survival improvement over time can be seen in male and female cases (Figure 3A, B, C, D and Table 3). At the beginning, males showed a higher 6-month RSR (76.2% vs. 69.3%, *p* < 0.001), but this advancement was reversed in the three subsequent decades (83.9% vs. 85.7%, 84.2% vs. 88.9% and 90.8% vs. 92.9% in males and females, respectively, in each decade, *p* < 0.0001). More interestingly, these changing survival disparities between sexes can be seen in most age groups but not the age group between five and nine years. Interestingly, all-cause survival showed an insignificant survival difference between sexes (*p* = 0.5795) (data not shown).

In terms of survival changes in each race over the past four decades, we observed that survival for Whites improved significantly, as demonstrated by the RSRs and Kaplan-Meier analyses (*p* < 0.0001) (Figure 3E, F, Supplementary Figure 1, and Table 4). Additionally, the increment in long-term survival is also more obvious than short-term survival in Whites. For instance, the 5-year RSRs increased from 22.1% to 38.4% to 61.0% to 72.2%, compared with the 6-month RSRs of 76.10%, 82.20%, 86.60% and 93.00%. In addition, the dramatic survival improvement can also be seen in Others, with the 5-year RSRs from 30.5% to 35.8% to 53.5% to 68.1%. Unfortunately, a modest survival improvement over time was observed in Blacks (*p* = 0.0144 for Kaplan-Meier analysis) (Figure 3G, H and Table 4). Furthermore, the improvement in both short-term and long-term RSRs remained modest over the four decades in Blacks.

**Figure 4:**
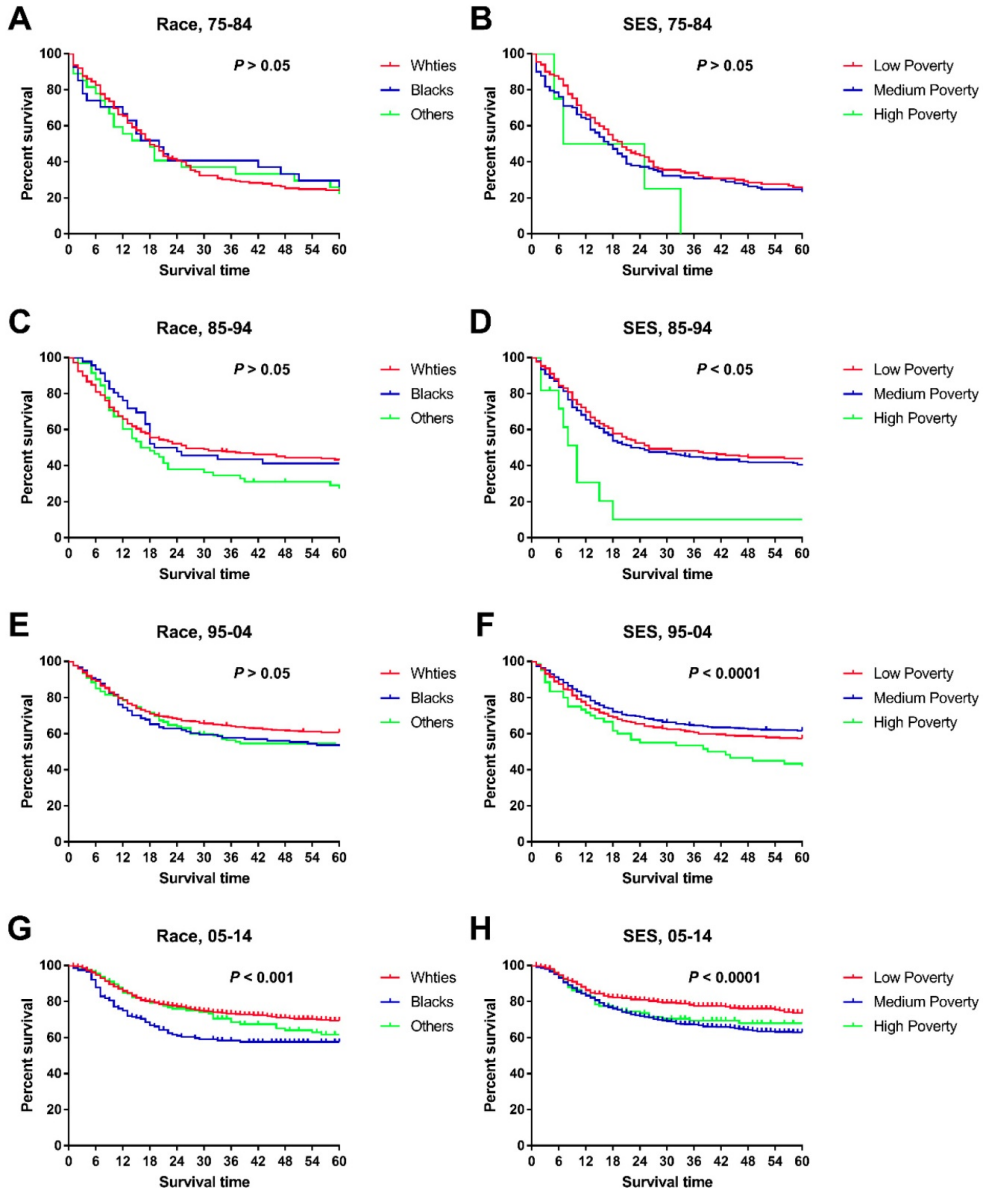
Trends in all-cause survival by Kaplan-Meier analyses by race (A, C, E, G.) and socioeconomic status (B, D, F, H.) in each decade in children patients diagnosed with chAML between 1975 and 2014 at the original nine SEER sites.

When we studied changes in the survival gap between Whites and Blacks, we found a widening racial survival gap over time, as demonstrated by the widening Kaplan-Meier curves (Figure 4). In addition, the survival gap among patients from different SES regions continued to widen over time, with a nonsignificant difference in the first decade and a significant difference in the last three decades (Figure 4 and Table 5). More interestingly, there was a difference in SES distribution between Whites and Blacks, with a higher proportion of Whites being categorized as low poverty than Blacks (33.85% vs. 22.01%), whereas Blacks had a higher proportion of medium-poverty patients (66.99% vs. 55.83%). As such, the widening survival gap among SES groups may indirectly help explain this racial survival disparity.

### Risk factors of death in chAML cases

To clarify the independent risk factors of death in chAML, Cox regression analyses were performed (Table 6). Age and race were proven to be significantly associated with patients' survival in univariate Cox regression. Multivariate Cox regression analyses demonstrate a race-specific risk difference, with a significantly higher risk of death in Blacks (HR = 1.245, *p* = 0.003), with Whites as a reference group. However, no risk difference was observed among age groups (HR = 0.965 and HR = 1.073 in cases aged between 5 and 9 years and cases aged between 10 and 14 years), compared with patients aged between 0 and 4 years.

## Discussion

This is the first study to demonstrate the rising incidence and increasing survival of acute myeloid leukemia in childhood (chAML) over four decades. More importantly, the survival gaps among races and SES groups spontaneously widened over time, which may be attributed to the racial difference in the distribution of SES status.

Although there is a high prevalence and mortality of chAML, its epidemiological trend has not been reported on the basis of a large sample. Based on our data, the incidence of chAML has increased steadily over the past four decades, from 5.766 to 6.615 to 7.478 to 7.607 per 1,000,000. Although the etiology for AML has not been clarified (except for the PML/RAR fusion gene in most M3 cases), several factors may be associated with the development of chAML: radiation, pesticide exposure during pregnancy, exposure to hair dyes, increased parental age, high birth weight, Down syndrome, Li-Fraumeni syndrome, Fanconi anemia, increased parental ages, antibiotic application and neurofibromatosis 23-32. A recent study demonstrated that the age of fathers in the U.S.A is increasing, with the mean paternal age increasing from 27.4 to 30.9 years over the past 44 years 33.

In addition, racial disparity in father age was also observed, with Asian fathers being the oldest and Black fathers being the youngest 33. Taken together, these facts showed that the higher paternal age of the father may help explain the increasing incidence of chAML, and the racial incidence disparity may partially be attributed to the racial disparity in parental age. In addition, the absolute patient number of chAML cases increased over time, and therefore, increased clinical awareness of AML in children should be addressed. In terms of sex, although males showed a lower incidence than females in the 1970s, the incidence in males climbed at a faster pace in subsequent decades. The sexual disparities may suggest the involvement of estrogen in chAML, in line with previous publications 34-38. This trend may provide an epidemiological clue for subsequent pathological studies when clarifying the effect of estrogen in AML. However, a previous study on childhood cancer, which was based on the California Cancer Registry, demonstrated that the incidence in males remained stable and the incidence in females increased significantly, which contradict our findings. This discrepancy may arise from the different data resources (California Cancer Registry vs. SEER registry) and patient enrollment criteria.

In terms of age, the highest incidence is found in patients aged between zero and four years, and the incidence increased at a faster rate. The highest incidence in newborn babies reflects the congenital nature of chAML and may provide epidemiological clues to identify its genetic cause. In terms of race, the incidence in Whites and Others remained relatively stable over time; however, the incidence in Blacks increased significantly. The aforementioned paternal age disparity among races may partially account for the racial incidence disparities. In addition, this racial disparity in incidence may also indicate racial differences in genetic background and lifestyle; therefore, further studies are needed to elucidate the molecular basis.

In terms of survival, the overall survival for chAML improved each decade, as indicated by RSRs and all-cause Kaplan-Meier analyses. This increasing survival trend was seen in every age group, sex, race and SES group. The standard treatment for chAML remains chemotherapy and hematopoietic stem cell transplantation 4. The first-line induction chemotherapy regimen for AML, except for the M3 subtype, has remained a DA regimen over the past three decades, and no successful addition of a novel chemical agent has been made 4. Improvements in survival for patients with chAML are attributed to the application of higher doses of anthracycline chemotherapy and supportive care for patients during the inevitable period of severe pancytopenia following effective chemotherapy 39, 40. Furthermore, the development of hematopoietic stem cell transplantation and second-line salvage chemotherapy may also contribute to the improved survival trend 40. In cases of acute promyelocytic leukemia (APL), the PML-RARα fusion gene has been proven to be the driver gene, and thereby, chemical agents targeting this gene show a therapeutic effect 41.

In terms of race, a significant survival disparity was observed among races, with the highest survival in Whites and the lowest survival in Blacks. The different responses toward chemotherapy and disparities in access to medication may be partially responsible for this racial survival disparity 42-44. Therefore, a tailored chemotherapy dosage and intensive care in Blacks to manage severe complications as well as healthcare policies to balance healthcare resource accessibility between Whites and Blacks should be reiterated. More importantly, the survival disparity between Whites and Blacks continued to widen over time. Previous studies have shown that SES may affect patient survival in many malignancies by affecting healthcare resource accessibility and screening 40, 45-47. Similarly, SES also affects survival in chAML, and this effect worsened over time, as indicated by the widening survival gaps. In the current study, we found that more Whites were categorized as low poverty than Blacks, and this different SES distribution partially explain this synchronous widening survival gap between races and SES subgroups.

Besides the novel results being here, the study was limited by following facts. First, as the patients are from the SEER database, the results and conclusions solely show trends in the nine registry sites, and caution is necessary when using the results to predict incidence and survival trends for patients in other regions. Second, the SEER-based study may cause bias if any miscalculation or underregistration occurred in the SEER database.

In summary, the present study showed increasing incidence as well as improved survival in chAML over time. Attention should be paid to patients aged between zero and four years and Blacks for their rapidly increasing incidence and lower survival. More importantly, the widening survival disparity in different races and SES groups was also observed, calling for a modification in the healthcare system to balance these disparities by providing well-covered medical resources. Studying the incidence and survival trend of chAML over time not only showed us changing epidemiological trends but also helped better design clinical trials by balancing these disparities.

## Supplementary Material

Supplementary figure and table.

## Figures and Tables

**Table 1:** Summary for children diagnosed with AML between 1975 and 2014, with number of patients and corresponding percentages in parentheses.

Categories	1975-2014	1975-1984	1985-1994	1995-2004	2005-2014	p value
	1490	288	342	424	436	
Age						
0-4	717(48.12%)	116(40.28%)	163(47.66%)	212(50.00%)	226(51.83%)	< 0.05
5-9	312(20.94%)	61(21.18%)	88(25.73%)	85(20.05%)	78(17.89%)	
10-14	461(30.94%)	111(38.54%)	91(26.61%)	127(29.95%)	132(30.28%)	
Sex						
Female	704(47.25%)	145(50.35%)	168(49.12%)	183(43.16%)	208(47.71%)	< 0.05
Male	786(52.75%)	143(49.65%)	174(50.88%)	241(56.84%)	228(52.29%)	
Race						
White	1061(71.21%)	225(78.13%)	249(72.81%)	299(70.52%)	288(66.06%)	< 0.05
Black	215(14.43%)	31(10.76%)	44(12.87%)	66(15.57%)	74(16.97%)	
Other	214(14.36%)	32(11.11%)	49(14.33%)	59(13.92%)	74(16.97%)	
SES						
Low	802(53.83%)	143(49.65%)	166(48.54%)	238(56.13%)	255(58.49%)	< 0.05
Medium	646(43.36%)	139(48.26%)	165(48.25%)	174(41.04%)	168(38.53%)	
High	42(2.82%)	6(2.08%)	11(3.22%)	12(2.83%)	13(2.98%)	
Vital status						
Alive	690(46.31%)	47(16.32%)	118(34.50%)	218(51.42%)	307(70.41%)	< 0.05
Dead	800(53.69%)	241(83.68%)	224(65.50%)	206(48.58%)	129(29.59%)	

Abbreviation: SES: socioeconomic status.

**Table 2:** Summary for 6-, 12-, 24-, 36-, 48- and 60-month RSRs, SEM and number of cases in the SEER 9 registry sites in each decade by calendar period.

RSR	Calendar Period											
	75-84			85-94			95-04			05-14		
	RSR(%)	SEM(%)	N	RSR(%)	SEM(%)	N	RSR(%)	SEM(%)	N	RSR(%)	SEM(%)	N
6-month												
ALL	74.2	2.6	287	84.8	2.0	341***	86.3	1.7	413***	91.8	1.4	411***
0-4	72.3	4.2	116	86.7	2.7	164***	86.6	2.4	208	91.5	1.9	220***
5-9	78.7	5.2	61	83.2	4.0	89***	87.7	3.7	81***	95.6	2.5	69***
10-14	73.6	4.2	110	82.9	4.0	88***	84.7	3.2	124**	90.2	2.7	122***
12-month												
ALL	59.1	2.9	287	66.2	2.6	341***	74.7	2.1	413***	84.2	1.8	411***
0-4	52.4	4.7	116	60.9	3.8	164***	73.2	3.1	208***	84.7	2.4	220***
5-9	75.4	5.5	61	74.2	4.6	89	77.8	4.6	81***	86.7	4.1	69***
10-14	57.3	4.7	110	67.9	5.0	88***	75.0	3.9	124***	81.9	3.5	122***
24-month												
ALL	37.3	2.9	287	47.3	2.7	341***	63.1	2.4	413***	75.2	2.2	411***
0-4	33.2	4.4	116	43.1	3.9	164***	63.1	3.4	208***	73.8	3.0	220***
5-9	53.6	6.4	61	54.0	5.3	89	67.9	5.2	81***	80.4	4.9	69***
10-14	32.8	4.5	110	48.4	5.4	88***	59.7	4.4	124***	74.9	4.0	122***
36-month												
ALL	29.2	2.7	287	44.1	2.7	341***	59.2	2.4	413***	72.3	2.3	411***
0-4	27.8	4.2	116	42.5	3.9	164***	60.8	3.4	208***	71.0	3.1	220***
5-9	41.9	6.4	61	49.5	5.3	89***	63.0	5.4	81***	76.9	5.3	69***
10-14	23.7	4.1	110	41.5	5.3	88***	54.1	4.5	124***	72.1	4.2	122***
48-month												
ALL	24.9	2.6	287	41.4	2.7	341***	57.0	2.4	413***	70.3	2.4	411***
0-4	26.9	4.2	116	38.8	3.8	164***	59.3	3.4	208***	71.0	3.1	220***
5-9	35.2	6.2	61	48.3	5.3	89***	61.7	5.4	81***	72.9	5.7	69***
10-14	17.3	3.6	110	39.2	5.2	88***	50.1	4.5	124***	67.7	4.4	122***
60-month												
ALL	22.4	2.5	287	39.6	2.7	341***	55.5	2.5	413***	68.3	2.5	411***
0-4	24.2	4.0	116	38.2	3.8	164***	58.3	3.4	208***	68.0	3.4	220***
5-9	33.5	6.1	61	47.2	5.3	89***	57.9	5.5	81***	72.9	5.7	69***
10-14	14.6	3.4	110	34.6	5.1	88***	49.3	4.5	124***	66.5	4.5	122***

Abbreviations: RSR, relative survival rate; SEM, standard error of the mean. *p < 0.01, **p < 0.001, and ***p < 0.0001 for comparisons with the preceding decade.

**Table 3:** Summary for 6-, 12-, 24-, 36-, 48- and 60-month RSRs, SEM and number of cases in the SEER 9 registry sites in each decade by sex and calendar period.

		Male			Female		
		RSR(%)	SEM(%)	N	RSR(%)	SEM(%)	N
75-84	6-month						
	ALL	76.2	6.0	51	69.3	5.7	65***
	0-4	80.0	7.3	30	77.4	7.5	31
	5-9	77.1	5.4	61	69.4	6.6	49***
	10-14	77.4	3.5	142	71.1	3.8	145***
85-94	6-month						
	ALL	83.9	2.8	174	85.7	2.7	167***
	0-4	85.2	4.0	81	88.0	3.6	83***
	5-9	81.6	5.5	49	85.0	5.6	40*
	10-14	83.9	5.6	44	81.8	5.8	44
95-04	6-month						
	ALL	84.2	2.4	233	88.9	2.3	180***
	0-4	85.7	3.2	118	87.9	3.5	90***
	5-9	86.1	5.3	43	89.5	5.0	38*
	10-14	80.6	4.7	72	90.4	4.1	52***
05-14	6-month						
	ALL	90.8	2.0	216	92.9	1.8	195***
	0-4	90.3	2.8	112	92.7	2.5	108***
	5-9	97.3	2.7	37	93.8	4.3	32**
	10-14	88.0	4.0	67	92.7	3.5	55***

Abbreviations: RSR, relative survival rate; SEM, standard error of the mean. *p < 0.01, **p < 0.001, and ***p < 0.0001 for comparisons with the Male group.

**Table 4:** Summary for 6-month RSRs, SEM and number of cases in the SEER 9 registry sites in each decade by race and calendar period.

		White			Black			Other		
	Year	RSR(%)	SEM(%)	N	RSR(%)	SEM(%)	N	RSR(%)	SEM(%)	N
95-84	6-month									
	ALL	76.10%	2.80%	225	66.80%	8.60%	31***	67.80%	8.40%	31***
	0-4	76.70%	4.40%	94	28.90%	17.20%	8***	64.30%	12.80%	14***
	5-9	80.90%	5.70%	47	77.80%	13.90%	9	60.00%	21.90%	5***
	10-14	72.60%	4.90%	84	78.60%	11.00%	14*	75.00%	12.50%	12
85-94	6-month									
	ALL	82.20%	2.40%	246	91.00%	4.30%	44***	91.60%	4.10%	48***
	0-4	85.60%	3.20%	124	93.90%	6.10%	16***	87.00%	7.00%	23
	5-9	75.00%	5.80%	56	94.50%	5.40%	18***	100.0%*	0.00%	13#
	10-14	81.80%	4.70%	66	80.00%	12.60%	10	91.30%	8.30%	12***
95-04	6-month									
	ALL	86.60%	2.00%	290	84.50%	4.50%	64***	86.00%	4.60%	57
	0-4	86.10%	2.90%	143	85.50%	6.1 0%	34	90.10%	5.50%	30***
	5-9	89.80%	3.90%	59	81.80%	11.60%	11**	80.00%	12.60%	10***
	10-14	85.20%	3.80%	88	84.20%	8.40%	19	82.40%	9.20%	17
05-14	6-month									
	ALL	93.00%	1.60%	271	86.60%	4.20%	67***	92.70%	3.20%	68
	0-4	91.90%	2.30%	147	87.70%	5.20%	40***	93.80%	4.30%	32**
	5-9	97.60%	2.40%	42	91.70%	8.00%	12**	93.30%	6.40%	15**
	10-14	92.70%	2.90%	82	80.00%	10.30%	15***	90.50%	6.40%	21

Abbreviations: RSR, relative survival rate; SEM, standard error of the mean. *p < 0.01, **p < 0.001, and ***p < 0.0001 for comparisons with the White group; #: t test is inapplicable for a zero value in SEM.

**Table 5:** Summary for 6-month RSRs, SEM and number of cases in the SEER 9 registry sites in each decade by SES and calendar period.

		Low-SES			Medium-SES			High-SES		
	Year	RSR(%)	SEM(%)	N	RSR(%)	SEM(%)	N	RSR(%)	SEM(%)	N
95-84	6-month									
	ALL	79.80%	3.40%	143	69.40%	3.90%	138***	50.00%	20.40%	6***
	0-4	79.80%	5.20%	59	65.00%	6.50%	55***	50.00%	35.40%	2***
	5-9	85.20%	6.80%	27	71.90%	7.90%	32***	100.00%	0.00%	2*
	10-14	77.20%	5.60%	57	72.60%	6.20%	51**	0.00%	0.00%	2***
85-94	6-month									
	ALL	84.40%	2.80%	166	85.40%	2.80%	164*	81.80%	11.60%	11
	0-4	85.10%	4.00%	80	87.80%	3.70%	81***	100.00%	0.00%	3***
	5-9	82.20%	5.70%	45	87.80%	5.10%	41***	33.30%	27.20%	3***
	10-14	85.20%	5.60%	41	78.60%	6.30%	42***	100.0%	0.00%	5#
95-04	6-month									
	ALL	84.00%	2.40%	231	89.00%	2.40%	171***	90.90%	8.70%	11***
	0-4	81.70%	3.70%	109	91.80%	2.80%	96***	100.0%	0.00%	3#
	5-9	86.70%	5.10%	45	88.60%	5.40%	35	100.0%	0.00%	1#
	10-14	85.70%	4.00%	77	82.50%	6.00%	40**	85.70%	13.20%	7
05-14	6-month									
	ALL	94.20%	1.50%	239	88.70%	2.50%	159***	83.90%	10.50%	13***
	0-4	96.10%	1.80%	125	84.70%	3.80%	91***	100.0%	0.00%	4#
	5-9	97.50%	2.50%	40	92.60%	5.00%	27***	100.0%	0.00%	2#
	10-14	89.20%	3.60%	74	95.10%	3.40%	41***	68.60%	18.60%	7***

Abbreviations: RSR, relative survival rate; SEM, standard error of the mean; SES: socioeconomic status. *p < 0.01, **p < 0.001, and ***p < 0.0001 for comparisons with the White group; #: t test is inapplicable for a zero value in SEM.

**Table 6:** Univariate and multivariate analyses for AML cases in children between 1975 and 2014.

	Univariate analysis			Multivariate analysis		
Characteristic	HR	95% CI	*P* value	HR	95% CI	P value
Age						
0-4	Reference	1		Reference	1	
5-9	1.017	0.889-1.163	0.810	0.965	0.797-1.168	0.712
10-14	1.135	1.012-1.274	0.031*	1.073	0.892-1.290	0.455
Sex						
Female	Reference	1		-	-	-
Male	1.006	0.909-1.114	0.903	-	-	-
Race						
White	Reference	1		Reference	1	
Black	1.252	1.084-1.446	0.022**	1.245	1.077-1.438	0.003**
Other	1.114	0.955-1.300	0.169	1.116	0.957-1.302	0.162
SES						
Low	Reference	1		-	-	-
Medium	1.088	0.976-1.214	0.130	-	-	-
High	1.181	0.961-1.451	0.114	-	-	-

Abbreviations: HR: hazard ratio; CI: confidential interval; SES: socioeconomic status. *p < 0.01 and **p < 0.001 for comparisons with the corresponding reference group.
