# Supplementary material for: The Epidemiological Trend of Acute Myeloid Leukemia in Childhood: a Population-Based Analysis
Source: J Cancer. 2019 Aug 27;10(20):4824–35. doi: 10.7150/jca.32326 (PMC6775523; doi:10.7150/jca.32326)
Supplement: Supplementary file 1 — Supplementary figure and table. [file jcav10p4824s1.pdf]

Supplementary figure 1.

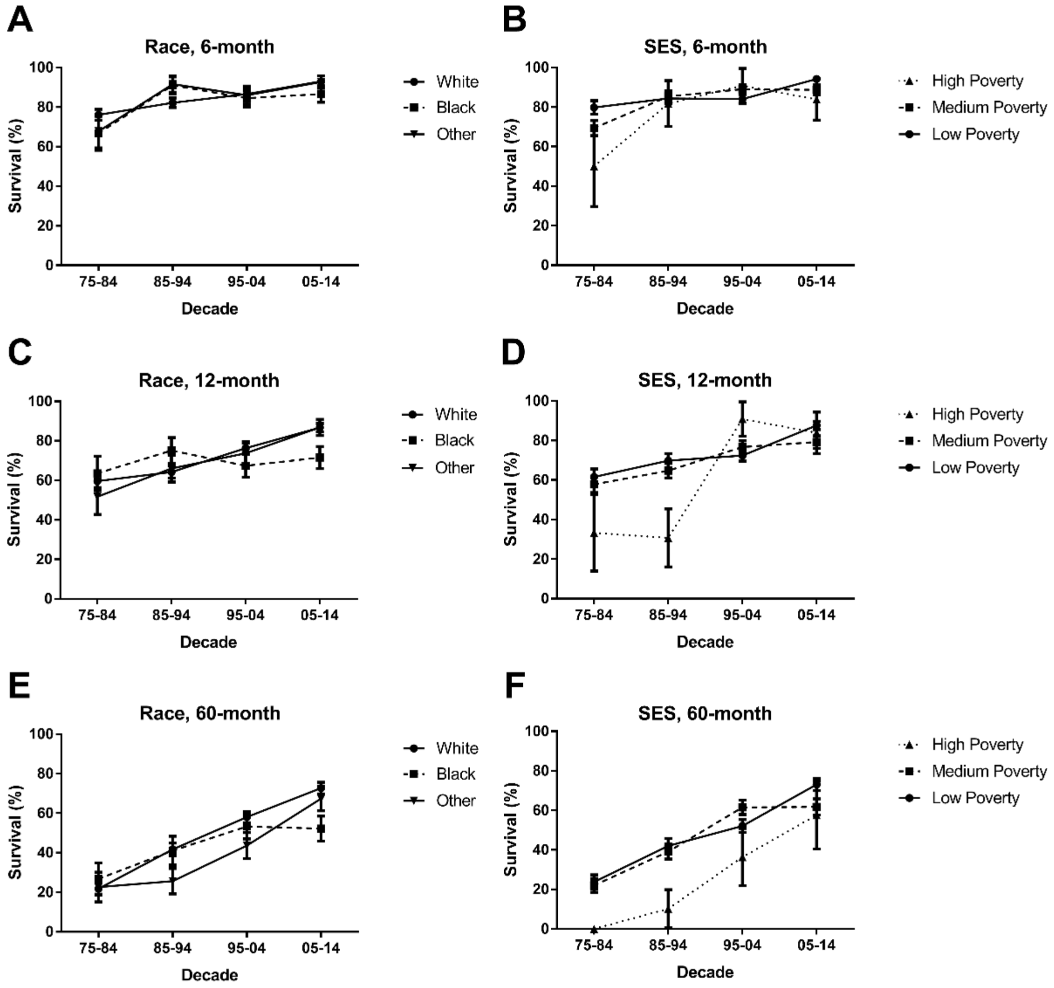

Supplementary Table 1. Summary for incidence data in the SEER 9 registry sites in each decade by age of diagnosis, race, SES and sex.

| Variables | Incidence per 1,000,000 by calendar period | | | |
|---|---|---|---|---|
| | 1975-1984 | 1985-1994 | 1995-2004 | 2005-2014 |
| Total | 5.766 | 6.615 | 7.478 | 7.607 |
| 0-4 | 7.279 | 9.16 | 11.471 | 11.9 |
| 5-9 | 3.819 | 5.253 | 4.531 | 4.142 |
| 10-14 | 6.269 | 5.558 | 6.625 | 6.984 |
| White | 5.616 | 6.206 | 7.169 | 6.986 |
| Black | 5.102 | 6.332 | 7.688 | 8.373 |
| Other | 8.458 | 9.639 | 8.884 | 9.393 |
| Low Poverty | 5.383 | 6.000 | 7.42 | 7.595 |
| Medium Poverty | 6.375 | 7.365 | 7.577 | 7.65 |
| High Poverty | 4.017 | 6.494 | 6.518 | 7.492 |
| Male | 5.598 | 6.586 | 8.297 | 7.762 |
| Female | 5.943 | 6.645 | 6.619 | 7.444 |
